# Evaluation of vegetation restoration effectiveness along the Yangtze River shoreline and its response to land use changes

**DOI:** 10.1038/s41598-024-58188-3

**Published:** 2024-03-31

**Authors:** Yinlan Huang, Xinyi Li, Dan Liu, Binyan Duan, Xinyu Huang, Shi Chen

**Affiliations:** School of Geography and Planning, Chizhou University, Chizhou, 247000 China

**Keywords:** Ecology, Environmental sciences

## Abstract

Assessing the effectiveness of vegetation restoration along the Yangtze River shoreline and exploring its relationship with land use changes are imperative for providing recommendations for sustainable management and environmental protection. However, the impact of vegetation restoration post-implementation of the Yangtze River Conservation Project remains uncertain. In this study, utilizing Sentinel-2 satellite imagery and Dynamic World land use data from pre- (2016) and post- (2022) Yangtze River Conservation Project periods, pixel-based binary models, transition matrices, and geographically weighted regression models were employed to analyze the status and evolution of vegetation coverage along the Yangtze River shoreline. The results indicated that there had been an increase in the area covered by high and high-medium vegetation levels. The proportion of vegetation cover shifting to better was 4201.87 km^2^ (35.68%). Hotspots of vegetation coverage improvement were predominantly located along the Yangtze River. Moreover, areas witnessing enhanced vegetation coverage experienced notable land use changes, notably the conversion of water to crops (126.93 km^2^, 22.79%), trees to crops (59.93 km^2^, 10.76%), and crops to built area (59.93 km^2^, 10.76%). Notably, the conversion between crops and built area emerged as a significant factor influencing vegetation coverage improvement, with average regression coefficients of 0.68 and 0.50, respectively. These outcomes underscore the significance of this study in guiding ecological environmental protection and sustainable management along the Yangtze River shoreline.

## Introduction

The Yangtze River, pivotal for national strategic development, possesses significant geographical advantages, abundant resources, thriving industries, a robust labor force, and a vast market^1,2^. It plays paramount role in fostering high-quality economic development, promoting green principles, constructing to China's beauty, and advancing ecological civilization^3,4^. However, economic growth pressures have severely degraded the Yangtze River Basin's environment^5^. The 20th National Congress emphasized ecological civilization construction, advocating green development and harmonious human-nature coexistence^6^. Since 2016, the Yangtze River Conservation proposal has made restoring the river's ecology crucial^7^. Anhui Province, as the link to the Yangtze River Economic Belt, has placed the restoration of the Yangtze River's ecological environment in a prominent position, focusing on greening the shoreline. It has delineated the "1515" shoreline classification and control red line (1 km, 5 km, and 15 km along the river), and has undertaken large-scale greening along both banks of the Yangtze River main stream^8–10^. Therefore, we evaluated the effectiveness of vegetation restoration within the 15 km defense line of the Yangtze River basin (Anhui section) and explored its response to changes in land use. This is of great significance for the in-depth implementation of the Yangtze River Conservation strategy and for safeguarding the ecological security along the Yangtze River in Anhui.

Vegetation plays a vital role in Earth's ecosystem, acting as a crucial link between humans and the environment, and maintaining ecological balance^11^. Vegetation cover serves as a primary indicator for assessing ecological equilibrium within a region^12^. Researchers have extensively investigated the correlation between vegetation cover and natural factors such as extreme weather, precipitation, and temperature^13–15^, alongside examining the impacts of human activities such as land use, urbanization, and ecological engineering on vegetation cover^16–18^. Scholars have particularly focused on studying the relationship between vegetation cover and ecosystem restoration in arid and semi-arid regions of China^19^, as well as in the Yellow-Huai-Hai and Yangtze River basins^20^, and the Loess Plateau^21^. They explore vegetation's role in geological disaster prevention and water conservation^22–25^, with studies indicating that enhancing vegetation cover contributes to improving natural environmental protection^26^. While researchers typically use remote sensing normalized difference vegetation index (NDVI) and statistical methods to analyze vegetation cover dynamics^27–31^, most studies have focused on overall trends and climate factors, neglecting detailed examinations of vegetation changes in different land types. The relationship between vegetation cover changes and land use evolution, as well as the spatiotemporal response of vegetation cover to land use changes, requires further investigation.

It is of great significance to scientifically grasp the effectiveness of vegetation restoration after the implementation of Yangtze River conservation for the restoration and protection of ecological environment and the promotion of high-quality development of the Yangtze River basin. However, in the six years since the implementation of the Yangtze River conservation strategy, a comprehensive investigation into the effectiveness of shoreline vegetation restoration was needed. Therefore, this study initially utilized Sentinel-2 remote sensing imagery in 2016 and 2022 to analyze the spatiotemporal variations in vegetation cover along the Yangtze River shoreline (15km) in Anhui province. Secondly, combined with Dynamic World land use data, the characteristics of land use changes in key vegetation change areas along the Yangtze River shoreline was explored. Thirdly, the geographical weighted regression model was used to reveal the spatiotemporal response of vegetation cover along the Yangtze River shoreline to land use change.

## Materials and methods

### Study area and data

**Figure 1:**
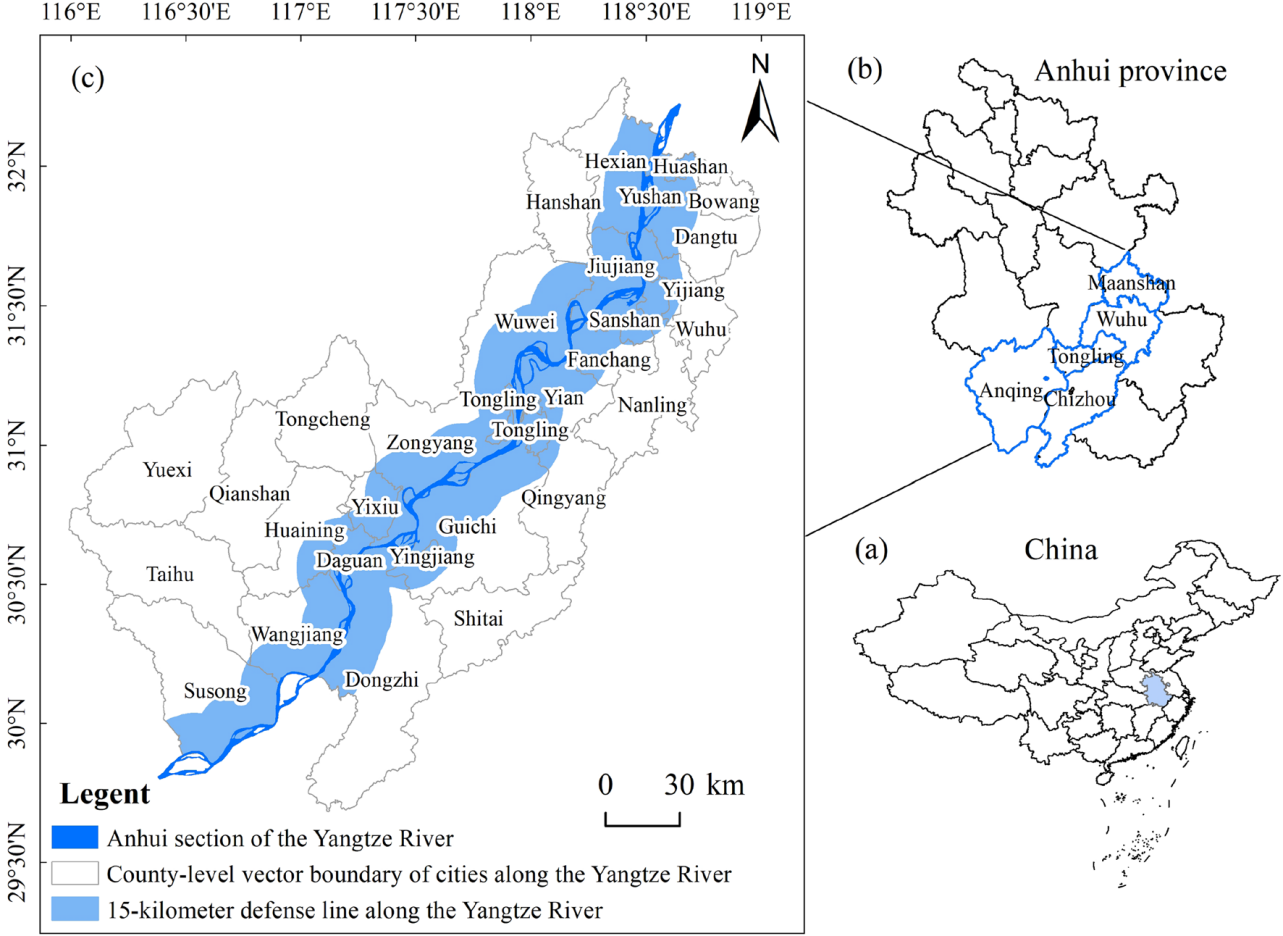
Overview of the study area.

The study area comprises a 15-kilometer buffer zone along the Yangtze River, encompassing Anqing City (Susong County, Wangjiang County, Huaining County, Daguang District, Yixiu District, Yingjiang District), Chizhou City (Dongzhi County, Guichi District, Qingyang County), Tongling City (Zongyang County, Tongguan District, Yi'an District), Wuhu City (Wuwei City, Fanchang County, Sanshan District, Jiujiang District, Yijiang District, Jinghu District, Nanling County, Wuhu County), and Ma'anshan City (He County, Dangtu County, Yushan District, Huashan District, Bowang District) within the geographical coordinates of 115°45' to 118°53'E and 29°21' to 32°04'N (Figure 1). The study area experiences a transition zone between warm temperate and subtropical climates, characterized by cold winters and hot summers, with distinct seasons and an average annual temperature ranging from 14-17°C. Precipitation, primarily concentrated in the summer, includes the plum rain season from late June to mid-July. The region features rivers, numerous lakes, and low hills on both riverbanks, with an elevation rising from south to north and a lower central portion^32^. Natural vegetation transitions from mixed forests of evergreen and deciduous broadleaf trees, with deciduous broadleaf trees in the north to predominantly evergreen broadleaf forests in the south of the Yangtze River plain. To assess vegetation restoration effectiveness in Anhui Province, this study focused on a 15 km buffer zone on both riverbanks, divided into 3 km × 3 km grid cells^33^, resulting in 1487 study units.

The vegetation data are in the form of the Normalized Difference Vegetation Index (NDVI), obtained from the Google Earth Engine cloud platform (https://earthengine.google.com). Initially, based on Sentinel-2 optical remote sensing data from 2016 and 2022, considering that extreme weather may cause changes in vegetation along the Yangtze River shoreline, this study selected cloud-free composite images with a 10-meter resolution during the long-term vegetation growth period (June to September). This was to avoid interference from floods, droughts, and other factors^34^. Subsequently, the NDVI for 2016 and 2022 was calculated. Finally, using a pixel-based binary model, the vegetation coverage for both periods was computed to characterize the vegetation status at different times. Land use data were sourced from the Dynamic World 10-meter resolution global land use dataset, also acquired through the Google Earth Engine cloud platform, which were derived from Sentinel-2 imagery with 10-meter resolution using deep learning methods. This study utilized land use data in 2016 and 2022, categorized into land use types including water, trees, grass, flooded vegetation, crops, shrubs, built areas, and bare ground^35^.

### Research methods

#### Pixel-based binary model

To evaluate the efficacy of vegetation restoration along the Yangtze River, and examine spatiotemporal dynamics of vegetation changes in relation to shifts in land use patterns, a pixel-based binary model was employed in this study to estimate vegetation coverage using NDVI data^36^. The calculation is as follows:

1 $${\text{FC}}=\frac{{\text{NDVI}}-{{\text{NDVI}}}_{{\text{soil}}}}{{{\text{NDVI}}}_{{\text{veg}}}-{{\text{NDVI}}}_{{\text{soil}}}}$$

In Equation (1), FC denotes the pixel vegetation coverage, NDVI represents the pixel normalized difference vegetation index, NDVI_veg_ signifies the NDVI value of pure vegetation pixels, and NDVI_soil_ indicates the NDVI value of pure bare soil pixels. Subsequently, vegetation coverage was determined using the pixel-based binary model, which partitioned the study area into non-vegetated and vegetated parts based on 95% and 5% thresholds, respectively, and assessed the contributions of these two components.

Drawing upon the "Soil Erosion Classification and Grading Standard" promulgated by the Ministry of Water Resources in 2008 and relevant research^24^ , which considered the specific conditions in the Yangtze River region of Anhui Province, vegetation coverage was stratified into six levels: non-vegetated area, <30% (low coverage), 30%~45% (medium-low coverage), 45%~60% (medium coverage), 60%~75% (medium-high coverage), and >75% (high coverage). This study employed the pixel binary model to monitor vegetation restoration^37^.

#### Hotspot analysis

The Getis-Ord Gi* method was employed to assess the clustering intensity of high or low values among the study objects within the neighborhood grid, thereby providing insight into the spatial correlation of each study object^38^. The calculation is as follows:

2 $${G}_{i}^{*}(d)=\frac{{\sum }_{j=1}^{n}{\omega }_{ij}{x}_{j}-\overline{X}{\sum  }_{j=1}^{n}{\omega }_{ij}}{S\sqrt{\frac{[n{\sum }_{j=1}^{n}{\omega }_{ij}^{2}-{({\sum }_{j=1}^{n}{\omega }_{ij})}^{2}]}{n-1}}}$$

In the formula, ${x}_{j}$ represents the area of the study object in grid j; ${\omega }_{ij}$ is the spatial weight matrix; $\overline{X }$ isthe average area of study objects in grid; n is the number of grid cells in the study area, and $S$ is the standard deviation. The ${G}_{i}^{*}$ calculation result is the Z-value, indicating cold spots if ${G}_{i}^{*}$ is a negative and significant value, and hot spots if ${G}_{i}^{*}$ is a positive and significant value. This study utilized hotspot analysis to investigate the characteristics of vegetation cover change and land use type change^39^.

#### Geographically weighted regression model

In this research, we applied a Geographically Weighted Regression (GWR) model to elucidate the relationship between changes in land use types and the restoration of vegetation cover, taking into account spatial autocorrelation and spatial heterogeneity^40^. The formula is presented below:

3 $$ y_{i} = \beta_{0} \left( {u_{i} ,v_{i} } \right) + \mathop \sum \limits_{k = 1}^{p} \beta_{k} \left( {u_{i} ,v_{i} } \right)x_{ik} + \varepsilon_{i} \qquad {\text{i}} = 1,2 \ldots \ldots ,{\text{n}} $$

In the formula, (${u}_{i},{v}_{i}$) is the coordinate of the i-th grid cell (e.g., latitude and longitude), ${\beta }_{k}$ (${u}_{i},{v}_{i}$) represents the k-th regression parameter at the i-th grid cell, which is a function of geographical location, ${\varepsilon }_{I}\sim N(0,{\sigma }^{2})$, ${\text{Cov}}({\upvarepsilon }_{{\text{i}}},{\upvarepsilon }_{{\text{j}}})$=$0$ (i ≠ j). In this investigation, the focal point revolves around the transition of vegetation cover within a grid from favorable to unfavorable conditions, or vice versa, constituting the dependent variable. Simultaneously, the independent variable comprises the transition of land use types within the corresponding grid. This study employed geographically weighted regression models to delineate the response dynamics of vegetation cover alterations in response to changes in land use types^41^.

To comprehensively assess the impact of alterations in land use types on the enhancement or degradation of vegetation cover along the Yangtze River shoreline, the transfer area of vegetation cover alterations within grid cells served as the dependent variable. The independent variable encompassed the area of land transformation types within the grid cells of vegetation cover alteration transfer zones. Utilizing the GWR model facilitated the computation of regression coefficients, where higher absolute values of these coefficients signify a more pronounced influence on vegetation alteration. Preliminary to executing the GWR model analysis, employing Ordinary Least Squares (OLS) is imperative to scrutinize multicollinearity issues among the independent variables.

## Results

### Temporal and spatial change analysis of vegetation cover

**Figure 2:**
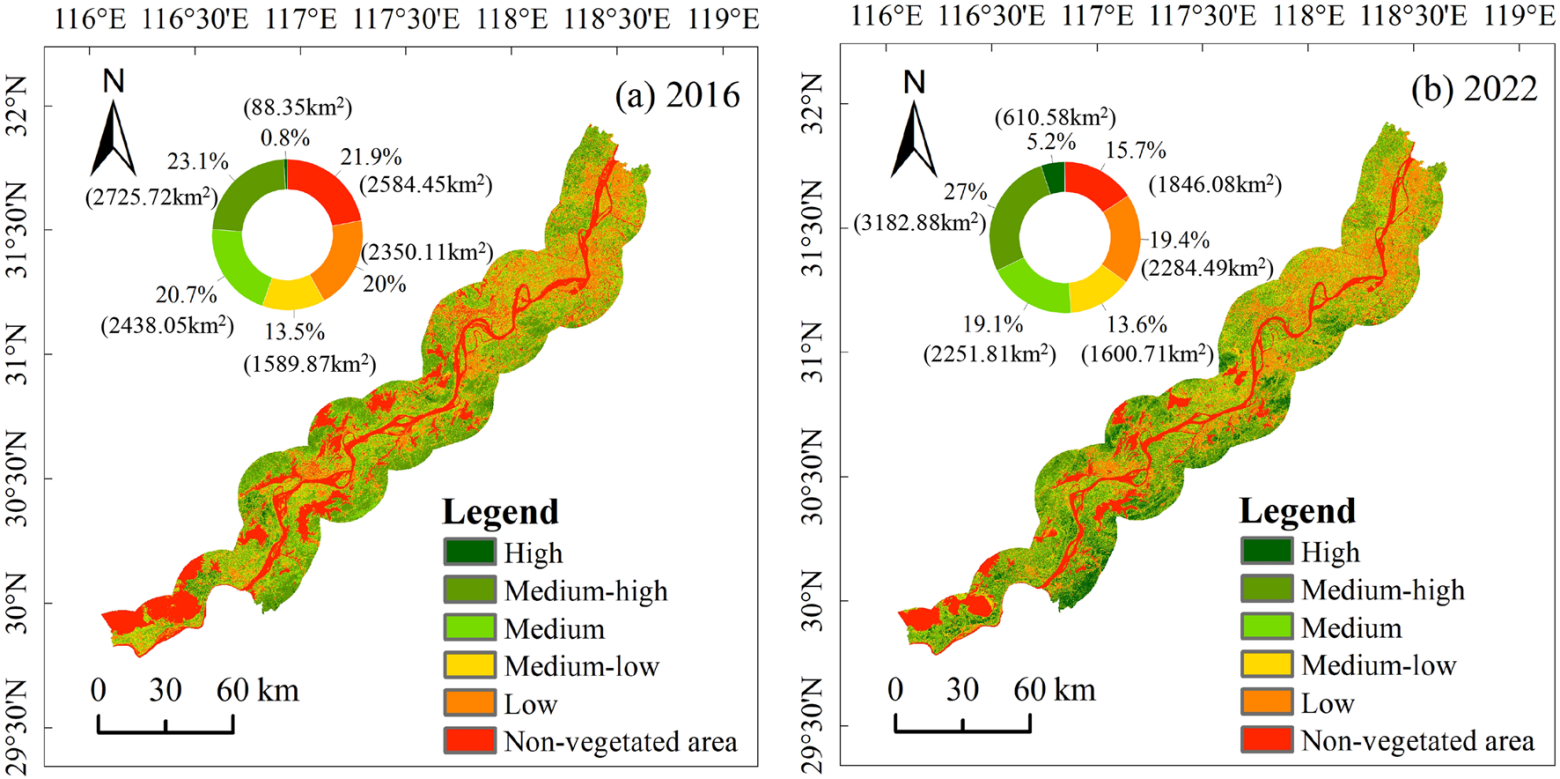
Spatial distribution of vegetation coverage levels before and after vegetation restoration.

The study examined the temporal and spatial dynamics of vegetation cover pre- and post-implementation of the Yangtze River protection project. In 2016, the study area predominantly exhibited three vegetation coverage tiers: medium-high, medium, and low. Notably, medium-high-level coverage accounted for 2725.72 km^2^ (23.1%), medium-level for 2438.05 km^2^ (20.7%), and low-level for 2350.11 km^2^ (20%). By 2022, vegetation coverage levels remained dominant in the medium-high, medium, and low categories, with 3182.88 km^2^ (27%), 2251.81 km^2^ (19.1%), and 2284.49 km^2^ (19.4%), respectively. The most significant changes occurred in the high and medium-high categories, witnessing increases of 515.77 km^2^ (4.4%) and 457.16 km^2^ (3.9%), respectively, compared to 2016. Additionally, a transition of 726.77 km^2^ (6.2%) transition from non-vegetated areas to vegetated areas was observed (Figure 2).

**Figure 3:**
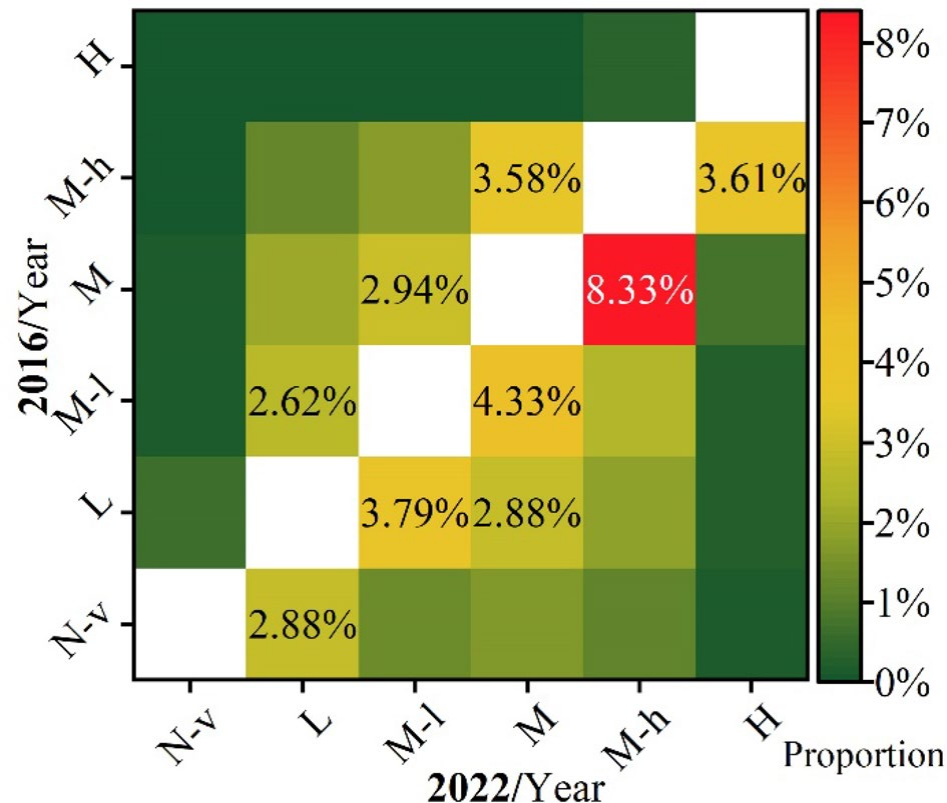
Proportion of changes in vegetation coverage levels.

Analysis of vegetation coverage transitions revealed that 48.68% remained unchanged, while 6043.73 km^2^ (51.32%) underwent alterations. Improved vegetation coverage transitions accounted for 4201.87 km^2^ (35.68%), primarily involving transitions from medium-level (M) to medium-high-level (M-h), medium-low-level (M-l) to medium-level, low-level (L) to medium-low-level, medium-high-level to high-level (H), low-level to medium-level, and non-vegetated (N-v) to low-level, with 980.99 km^2^ (8.33%), 509.92 km^2^ (4.33%), 446.33 km^2^ (3.79%), 425.13 km^2^ (3.61%), 339.16 km^2^ (2.88%) and 339.16 km^2^ (2.88%), respectively. Decreased vegetation coverage accounted for 1841.85 km^2^ (15.64%), primarily transitioning from medium-high-level to medium-level, medium-level to medium-low-level, and medium-low-level to low-level, with 421.6 km^2^ (3.58%), 346.23 km^2^ (2.94%), and 308.55 km^2^ (2.62%), respectively (Figure 3).

Within 15km along the Yangtze River shoreline, vegetation coverage levels in 2016 and 2022 were predominantly classified into three levels: medium-high, medium, and low. The respective areas were 7513.88 km^2^ (63.8%) and 7719.18 km^2^ (65.5%). Following the implementation of the Yangtze River conservation strategy, there was a notable improvement in vegetation coverage, with 4201.87 km^2^ (35.68%) transitioning positively and 1841.85 km^2^ (15.64%) transitioning negatively. This indicates a significant enhancement in vegetation restoration effectiveness.

**Figure 4:**
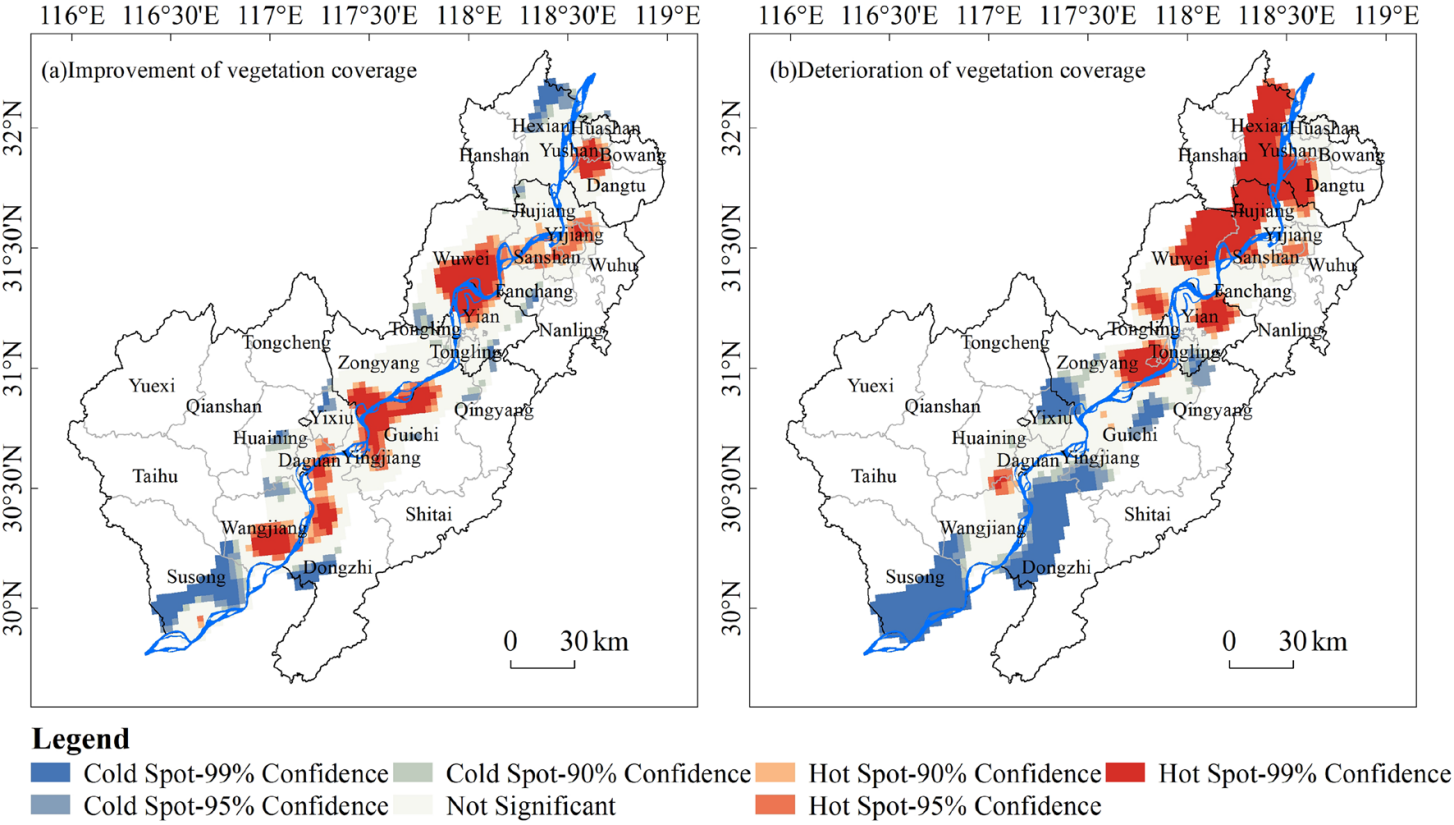
Spatial distribution of transition hot spots (improvement) and cold spots (deterioration) in vegetation coverage along the Yangtze River Shoreline.

Regarding the spatial distribution of vegetation coverage transitions, areas with increased vegetation coverage exhibited a hotspot pattern, primarily located along the Yangtze River in areas such as Wangjiang County, Dongzhi County, Guichi District, Zongyang County, Yixiu District, and Wuwei City (Figure 4a). In contrast, areas with a decreased vegetation coverage displayed a pattern of hot in the north and cold in the south with hotspots in regions like He County, Yushan District, Dangtu County, Jiujiang District, Wuwei City and parts of Zongyang County and Yi'an District in Tongling City (Figure 4b).

### Characteristics of land use changes in key vegetation change areas

**Figure 5:**
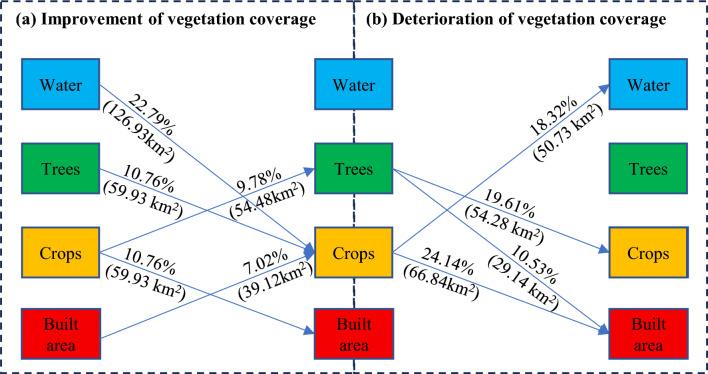
Proportion of land use type transitions in key vegetation change areas along the Yangtze River Shoreline.

In regions experiencing positive shifts in vegetation coverage, land use transformations primarily involved conversions from water to crops, trees to crops, crops to built area, crops to trees, and built area to crops. These transitions accounted for 126.93 km^2^ (22.79%), 59.93 km^2^ (10.76%), 59.93 km^2^ (10.76%), 54.48 km^2^ (9.78%), and 39.12 km^2^ (7.02%) of the total area undergoing positive vegetation coverage transitions, respectively (Figure 5a). Conversely, areas with negative vegetation coverage transitions, witnessed changes primarily from crops to built area, trees to crops, crops to water, and trees to built area, accounting for 66.84 km^2^ (24.14%), 54.28 km^2^ (19.61%), 50.73 km^2^ (18.32%), and 29.14 km^2^ (10.53%) of the entire area of negative vegetation coverage transitions, respectively (Figure 5b). It is evident that land use type transitions primarily involved crops, built area, water, and trees.

**Figure 6:**
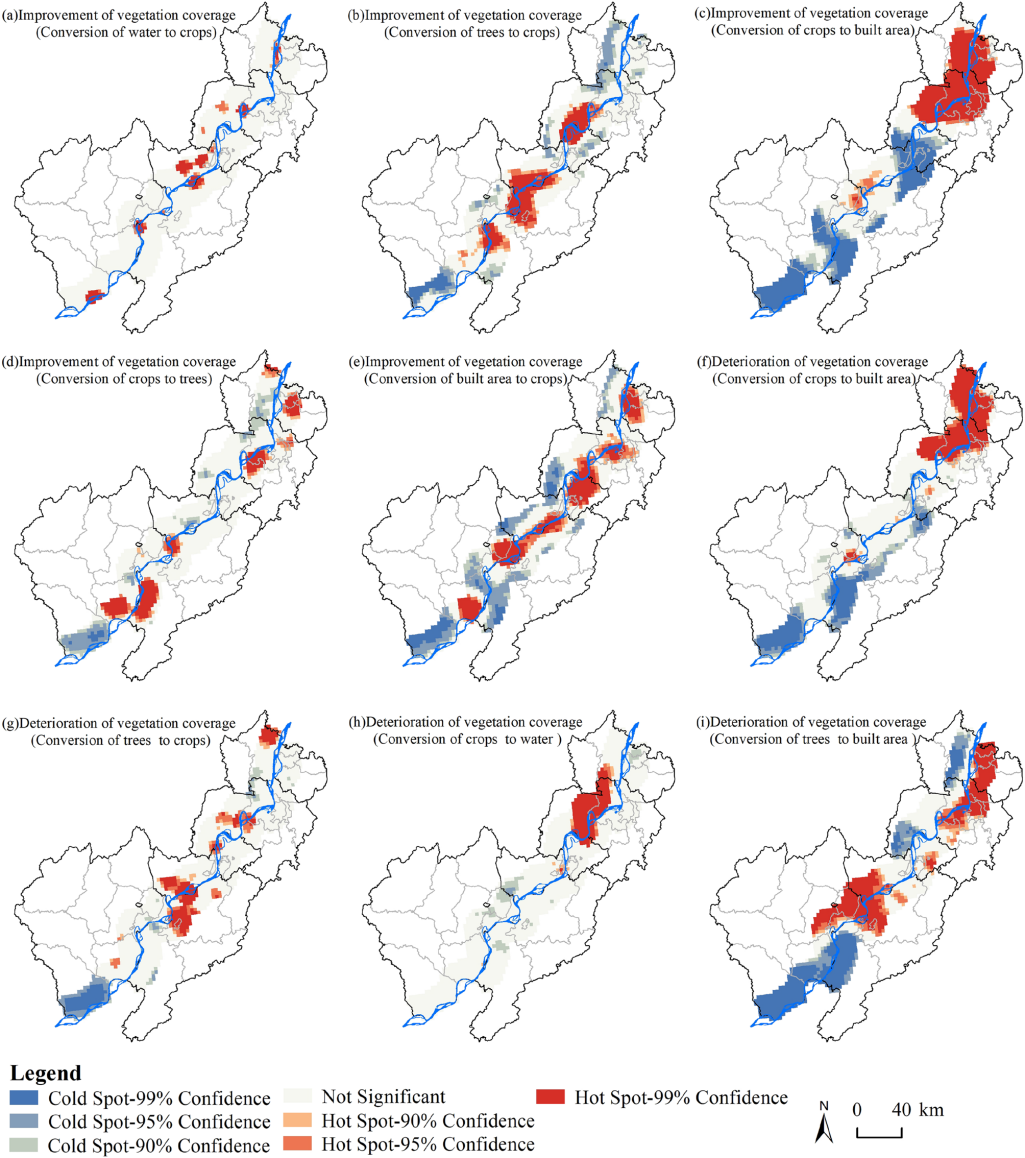
Spatial distribution of hotspots and coldspots of the main land use type transition in key vegetation change areas along the Yangtze River Shoreline.

Spatially, the distribution of improved vegetation coverage: hotspots in the conversion of water to crops conversions along both sides of the Yangtze River's periphery, particularly in Susong County, the northwestern parts of Dongzhi County, the bordering areas of Guichi District and Zongyang County, and certain areas within Wuhu City and Ma'anshan City (Figure 6a). Concentrated hotspots for trees to crops conversions are observed in the bordering areas of Anqing City, Chizhou City, and Tongling City, as well as in the bordering regions of Wuhu City and Tongling City (Figure 6b). The transition from crops-to-built areas exhibits a pattern of hotspots in the northern region particularly in Wuhu City and Ma'anshan City, with coldspots in the southern region (Figure 6c). Hotspots for transitions crops-to-trees transitions, hotspots were distributed along the Yangtze River shoreline, particularly in the southern parts of Wangjiang County, Dongzhi County, the bordering area of Yingjiang District, Guichi District, and Zongyang County, as well as in certain parts of Wuhu City's Sanshan District and Ma'anshan City's Yushan District (Figure 6d). Hotspots in crops-to-water transitions were located along both sides of the Yangtze River, especially in the areas of Wangjiang County, Guichi District, Zongyang County, Yi'an District, Jiujiang District, and Huashan District (Figure 6e).

In areas with negative vegetation coverage transitions, hotspots in crops-to-built area were mainly found in large parts of Wuhu City and Ma'anshan City (Figure 6f). For trees-to-crops transitions, hotspots were concentrated in the northern parts of Guichi District and the southern parts of Zongyang County (Figure 6g). In the case of crops-to-water transitions, hotspots were primarily located at the junction of Sanshan District and Wuwei City (Figure 6h). For trees-to-built area transitions, hotspots were mainly observed in large parts of the bordering regions of Anqing City, Chizhou City, and Tongling City, as well as in some areas along the border of Wuhu City and Ma'anshan City (Figure 6i).

### Spatiotemporal response of vegetation coverage to land use changes

**Figure 7:**
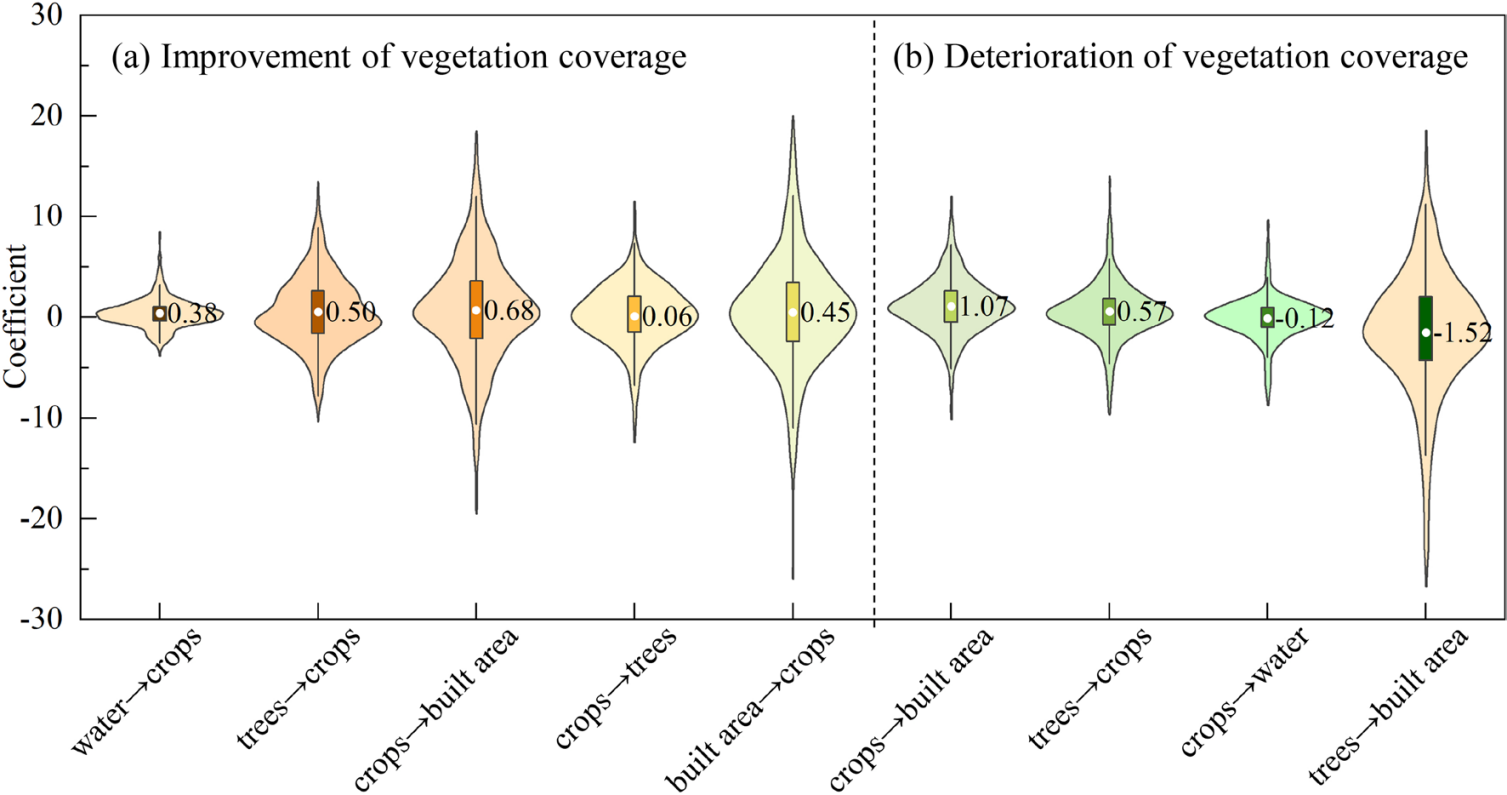
Regression coefficients of land use transition types on vegetation coverage changes.

In terms of vegetation cover improvement, the GWR goodness of fit (*R*^2^) was 0.55, and Akaike's Information Criterion corrected (AICC) was 44293.79. Land use transfer types exhibited a positive influence on vegetation cover improvement overall. The average regression coefficients for the conversion of crops to trees, water to crops, built area to crops, trees to crops, and crops to built area increased sequentially, which were 0.06, 0.38, 0.45, 0.50, and 0.68, respectively. Notably, the conversions of crops to built area and trees to crops had a significant impact (Figure 7a).

Regarding the deterioration of vegetation cover, the GWR goodness of fit (*R*^2^) was 0.63, and AICC was 43040.13. Conversions of crops to built area and trees to crops demonstrated a positive effect on the deterioration of vegetation cover, with average regression coefficients of 1.07 and 0.57, respectively. Conversely, conversions of trees to built area and crops to water exhibited a negative impact on the deterioration of vegetation cover, with average regression coefficients of -1.52 and -0.12, respectively (Figure 7b).

**Figure 8:**
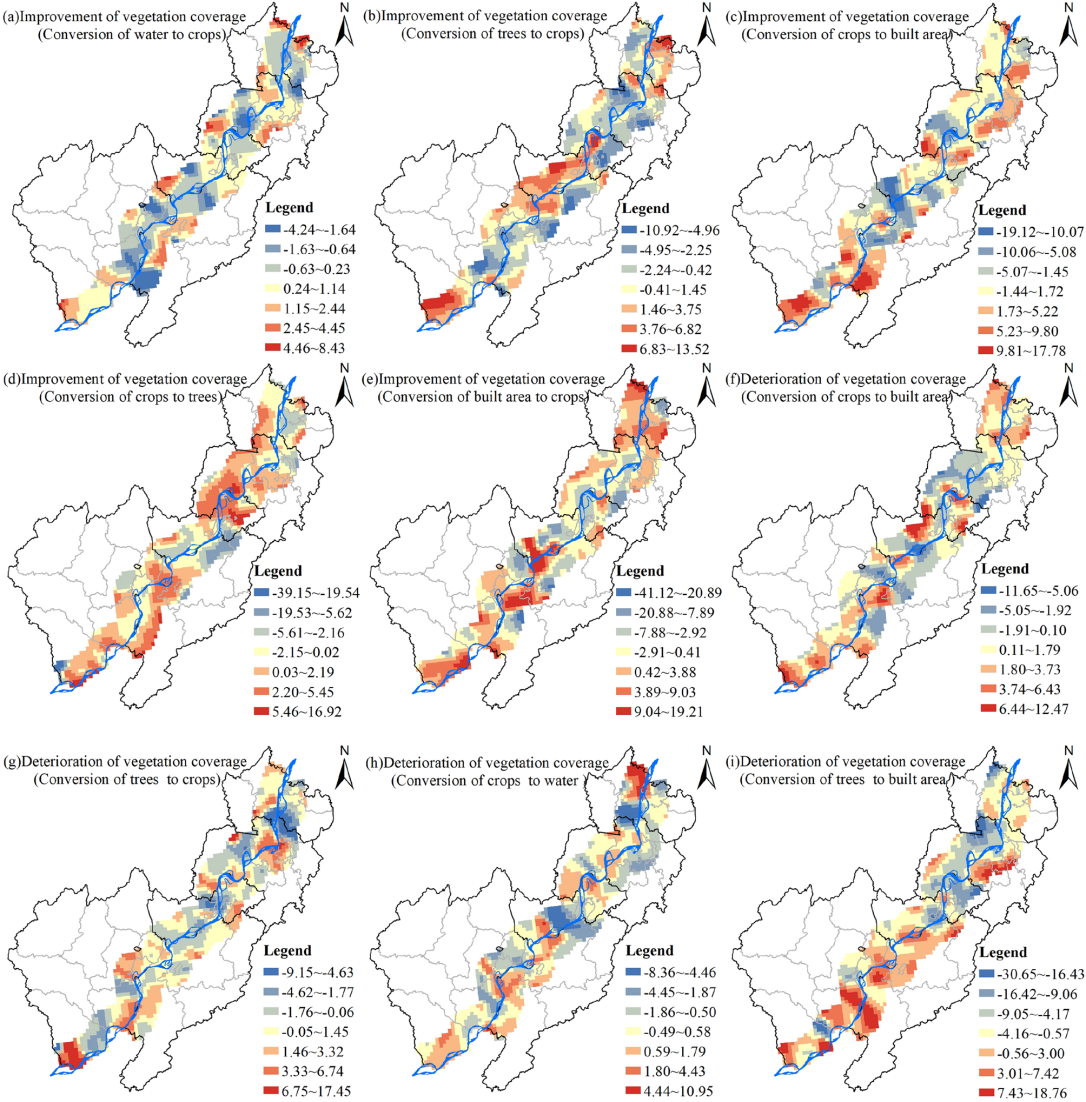
Spatial Distribution of the Impact of Land Use Type Transitions along the Yangtze River Shoreline on Vegetation Coverage Improvement (Deterioration).

In terms of spatial pattern differences, the regression coefficients for vegetation cover improvement associated with conversions from water to crops, trees to crops, crops to built area, crops to trees, and built area to crops ranged from -4.24 to 8.43 (Figure 8a), -10.92 to 13.52 (Figure 8b), -19.12 to 17.78 (Figure 8c), 39.15 to 16.92 (Figure 8d), and -41.12 to 19.21 (Figure 8e), respectively. Positive high-value areas for crops to built area were observed in southern Susong County, western Dongzhi County, and parts of Wuhu and Ma'anshan, while negative low-value areas appeared at the border of Chizhou and Tongling cities. Similarly, positive high-value areas for trees to crops were found in southern Susong County, large parts of Tongling City, and some areas of Ma'anshan, while negative low-value areas occurred in parts of Chizhou and Wuhu cities. It's evident that changes in crops to built area and trees to crops are significant factors influencing the improvement of vegetation cover.

Regarding the deterioration of vegetation cover, the regression coefficients for crops to built area, trees to crops, crops to water, and trees to built area ranged from -11.65 to 12.47 (Figure 8f), -9.15 to 17.45 (Figure 8g), -8.36 to 10.95 (Figure 8h), and -30.65 to 18.76 (Figure 8i), respectively. Positive high-value regions were observed in the western part of Dongzhi County to the northwest of Chizhou City, at the border of Tongling City with Wuhu City, among others. Specifically, positive high-value areas for trees to built area occurred in large parts of the border between Dongzhi County and Wangjiang County, along the Yangtze River in Dongzhi County and Guichi District, and in some areas of Wuhu City, while negative low-value areas were present at the junction of Ma'anshan City with Wuhu City and Tongling City with Wuhu City. Positive high-value areas for crops to built area were observed in the western part of Dongzhi County to the northwest of Chizhou City, at the border of Tongling City with Wuhu City, among others, while negative low-value areas were found in large parts of Wuhu City and Guichi District. Thus, conversions from trees to built area and crops to built area are significant influencing factors leading to the deterioration of vegetation cover.

## Discussion

### The influence factor of vegetation cover changes

Large-scale and long-term vegetation cover changes are influenced by natural environmental factors such as topography, geomorphology, and climate change, while small-scale and short-term vegetation cover changes are significantly impacted by human activities such as ecological engineering and urbanization^42^. Over the long term, 81.5% of vegetation shows a significant positive correlation with precipitation, and 82.2% exhibits a significant positive correlation with temperature^43^. In the middle and lower reaches of the Yangtze River, human activities have a greater impact on vegetation compared to climate change^44^. Although climate change has a greater impact on the area of vegetation than human activities, its significance is not as pronounced. The influence of human activities on vegetation change is closely related to local ecological policies ^45^. For example, the implementation of ecological afforestation projects in the Yangtze River Basin has achieved significant results in vegetation restoration. By 2013, the forest area reached 6.85 × 10^5^ km^2^, and vegetation cover increased by 15.86% over 12 years ^46^. Under the background of the Yangtze River Protection, Anhui Province has actively implemented ecological greening policies to restore the ecological environment along the Yangtze River, improving the vegetation cover. Our study found that 4201.87 km^2^ of vegetation cover levels have shifted towards improvement, mainly reflected in the transition from medium-level to medium-high-level, medium-low-level to medium-level.

Land use changes related to ecological restoration projects are the main driving force for vegetation improvement in the Yangtze River Basin^47^. The conversion of land use types to trees, crops, and grassland is the primary reason for increasing vegetation cover, with contributions rates of 60.66%, 20.93%, and 18.41% respectively^48^. The conversion of land use types to trees plays a crucial role in vegetation restoration and protection. It was noteworthy that 39.12 km^2^ of built area has been converted into crops, leading to an increase in vegetation cover along the Yangtze River in Anhui. This was mainly due to urbanization promoting the migration of rural populations to cities, releasing built area, which was then reclaimed or restored for crops through land consolidation or reclamation^49^. The implementation of ecological projects such as returning farmland to forests and afforestation of millions of hectares in Anhui Province has increased forest area. For instance, between 2000 and 2020, the greening areas of Anqing and Chizhou increased by 169.30 km^2^ and 75.98 km^2^ respectively, contributing significantly to vegetation restoration^50^. The conversion of other land use types to construction land is the most important driver of vegetation change caused by human activities^51^. After the implementation of the Yangtze River Protection in the 15km buffer zone along the Yangtze River in Anhui Province, with the development of urbanization, 126.77 km^2^ of crops was converted to built area, of which 59.93 km^2^ contributed to the improvement of vegetation, and 66.84 km^2^ contributed to the deterioration of vegetation^52^. Under the policy drive of farmland reclamation,114.21 km^2^ of trees was converted to crops, of which 59.93 km^2^ contributed to the improvement of vegetation, and 54.28 km^2^ contributed to the deterioration of vegetation. When land use types shift from crops to built area, vegetation cover usually decreases due to land development, deforestation, land surface hardening, and construction activities, all of which lead to vegetation destruction and reduction^53^. However, in this study, vegetation cover may increase, as research indicated that the number of urban green spaces in almost all Chinese cities has increased^54^. This increase is more significant in cities with better economic development and larger urban areas, as there is an increase in green space, parks, green belts, as well as trees and shrubs^55^.

### Limitations

The paper research still has some shortcomings. Extreme weather events such as floods and droughts in the Yangtze River Basin may affect vegetation monitoring^56,57^. Fortunately, hydraulic engineering projects like the Three Gorges Dam can mitigate extreme floods and regulate downstream flow during the dry season, to some extent avoiding damage to vegetation cover^58,59^. Additionally, this study focused on monitoring vegetation during the long growing season. Short-term vegetation changes caused by floods and droughts can be eliminated through the maximum vegetation index value processing^60^. Continuous monitoring of vegetation cover along the Yangtze River at an annual scale is limited due to the cloudy and rainy conditions in the Anhui Province along the river, restricting the acquisition of high-quality annual-scale Sentinel-2 remote sensing data. Therefore, this study opted to evaluate the vegetation cover using remote sensing data from before (2016) and after (2022) the implementation of the Yangtze River Protection Program. Regarding land use data selection, the study utilized nearly real-time updated Dynamic World data, but the confusion between wetlands, grasslands, and bare land may introduce some errors into the research results. In terms of vegetation cover spatiotemporal monitoring methods, the paper solely employed the transition matrix method. In future research, the use of spatiotemporal change intensity analysis could be considered to analyze the transition trends and their impacts between different levels of vegetation cover from relative and absolute perspectives.

## Conclusions

After the implementation of the Yangtze River protection strategy, the vegetation coverage along the Anhui section exhibited three dominant levels: medium-high, medium, and low. Nevertheless, there was a noteworthy increase in vegetation coverage proportions at the high and medium-high levels, indicating enhancement in overall vegetation coverage. The shift from non-vegetated to vegetated land likely stems from the successful execution of vegetation protection policies.

In regions experiencing positive transitions in vegetation coverage, notable shifts in land use types included conversions from water to crops, trees to crops, crops to built areas, crops to trees, and built areas to crops. Conversely, areas with negative transitions in vegetation coverage, witnessed major land use changes involving conversions from crops to built areas, trees to crops, crops to water, and trees to built areas. These alterations in land use types underscore the intricate interplay between vegetation coverage and land use changes, particularly their impact on positive and negative vegetation transitions.

Land use categories such as crops, trees, and built areas significantly influenced vegetation coverage changes. Transitions from crops to built areas and from trees to crops were pivotal factors driving positive vegetation transitions, while transitions from trees to built areas and from crops to built areas were primary contributors to negative vegetation transitions.

## Data Availability

All data generated or analyzed during this study are included in this published article.
